# Defining abnormal cold sensitivity using the Cold Intolerance Symptom Severity questionnaire: a population study

**DOI:** 10.1177/1753193421996221

**Published:** 2021-03-12

**Authors:** Albin Stjernbrandt, Ingrid Liljelind, Tohr Nilsson, Jens Wahlström

**Affiliations:** Section of Sustainable Health, Umeå University, Umeå, Sweden

**Keywords:** Hand, cold exposure, occupational exposure, hand-arm vibration, nerve injury, Sweden

## Abstract

Cold sensitivity, a common and disabling sequela of hand injury, can be assessed using the Cold Intolerance Symptom Severity (CISS) questionnaire, rating symptoms on a scale from 4 to 100. The primary objective of this study was to define a clinical cut-off for abnormal cold sensitivity based on the CISS score in a healthy working-age population. The secondary objective was to investigate how age, gender and previous injuries and diseases influence CISS scoring. In this study, 1239 out of 1582 selected healthy subjects of working age living in northern Sweden completed the questionnaire, yielding a response rate of 78%. The 95th percentile for the CISS score was 49.5 for men and 53.0 for women. The effects of age, gender and previous injuries and diseases were minor and not considered clinically relevant. The results support that a CISS score above 50 should be considered as abnormal cold sensitivity.

**Level of evidence:** III

## Introduction

Cold sensitivity, or cold intolerance, was defined by Campbell and Kay (1998) as a collection of acquired symptoms, resulting in an abnormal aversion to cold with pain, altered sensibility, stiffness or colour changes. It has been reported as a sequela to different kinds of traumatic hand injuries (Lithell et al., 1997), cold injuries (Hutchison, 2014) and hand–arm vibration (HAV) syndrome (Carlsson et al., 2010b). Subjects with cold sensitivity often report a major negative impact on quality of life and work ability (Lithell et al., 1997), but workers’ compensation claims are often rejected, partly because of the lack of objective measures (Graham and Schofield, 2008). The diagnosis of cold sensitivity is usually based solely on the symptoms reported by the individual.

The description of symptoms can be supported by a standardized questionnaire; the first validated questionnaire that was widely adopted was the Cold Sensitivity Severity scale (McCabe et al., 1991), and it was subsequently modified into the Cold Intolerance Symptom Severity (CISS) score (Irwin et al., 1997). The CISS score assesses six main items with several subsets of questions to evaluate both the experienced symptoms (such as pain, numbness, stiffness, reduced grip strength, swelling and skin colour changes); actions taken to prevent or ease symptoms (e.g. using gloves or staying indoors), as well as the consequences for activities of daily living (e.g. undertaking domestic chores or working). The score is summarized on a scale ranging from 4 to 100, with higher scores indicating worse symptoms (Table S1, available online).

In the original article, the authors arbitrarily grouped the scores into four ranges (mild, 4–25; moderate, 26–50; severe, 51–75; and extremely severe, 76–100) (Irwin et al., 1997). More recently, a clinical cut-off of 30 was suggested for pathological cold sensitivity, based on the mean + 2 standard deviations (SD) of a sample of 68 healthy subjects from the Netherlands, using a Dutch version of the CISS questionnaire, and excluding subjects with injury or previous surgery to the upper extremity or Raynaud’s phenomenon (Ruijs et al., 2006). The CISS questionnaire has also since been translated to Swedish, and analyses of the reliability and validity performed in this language (Carlsson et al., 2008). For the Swedish version, a CISS score exceeding 50, instead of 30, was established as a cut-off, based on the 95th percentile of a cohort of 81 randomly selected healthy volunteers, where previous hand injury was the sole exclusion criterion (Carlsson et al., 2010a). The large discrepancy between the two suggested thresholds has introduced difficulty in discerning what could be considered an abnormal state of cold sensitivity. There is therefore a need for a larger source of reference material to determine a suitable cut-off score for pathological cold sensitivity, where effects of age and gender, as well as previous diseases and injuries that might influence the scoring are taken into account.

The primary objective of this study was to define a clinical cut-off for abnormal cold sensitivity based on the CISS score in a large healthy working-age population. The secondary objective was to investigate how different parameters, such as age, gender and previous injuries and diseases, influence CISS scoring.

## Methods

### Study design

Participants were drawn from the national Swedish population register, as part of a questionnaire-based research project called Cold and Health in Northern Sweden (CHINS), targeting those aged 18 to 70, and living in the four northernmost counties in Sweden. Two case-control studies on cold sensitivity and Raynaud’s phenomenon were performed (Stjernbrandt et al., 2018, 2019), and the healthy controls from these studies formed the current study population, utilizing original data. The participants were asked to respond to two questionnaires about cold-related symptoms. The first questionnaire (CHINS1) contained 45 questions, including height, weight, tobacco habits and previous diseases. The second questionnaire (CHINS2) contained an additional 45 questions, beginning with the Swedish version of the CISS questionnaire (Carlsson et al., 2010a), and continuing with items regarding previous injuries, current medication, as well as exposure to cold climate and HAV. Those who reported cold sensitivity or Raynaud’s phenomenon in the first questionnaire were excluded. The data collection has previously been described in detail (Stjernbrandt et al., 2017; 2018). Written informed consent was obtained from all subjects and the study protocol was approved by the Regional Ethical Review Board situated at Umeå University (DNR 2014-286-31 M, 2015-24-32 M and 2015-255-32 M).

**Figure 1.**
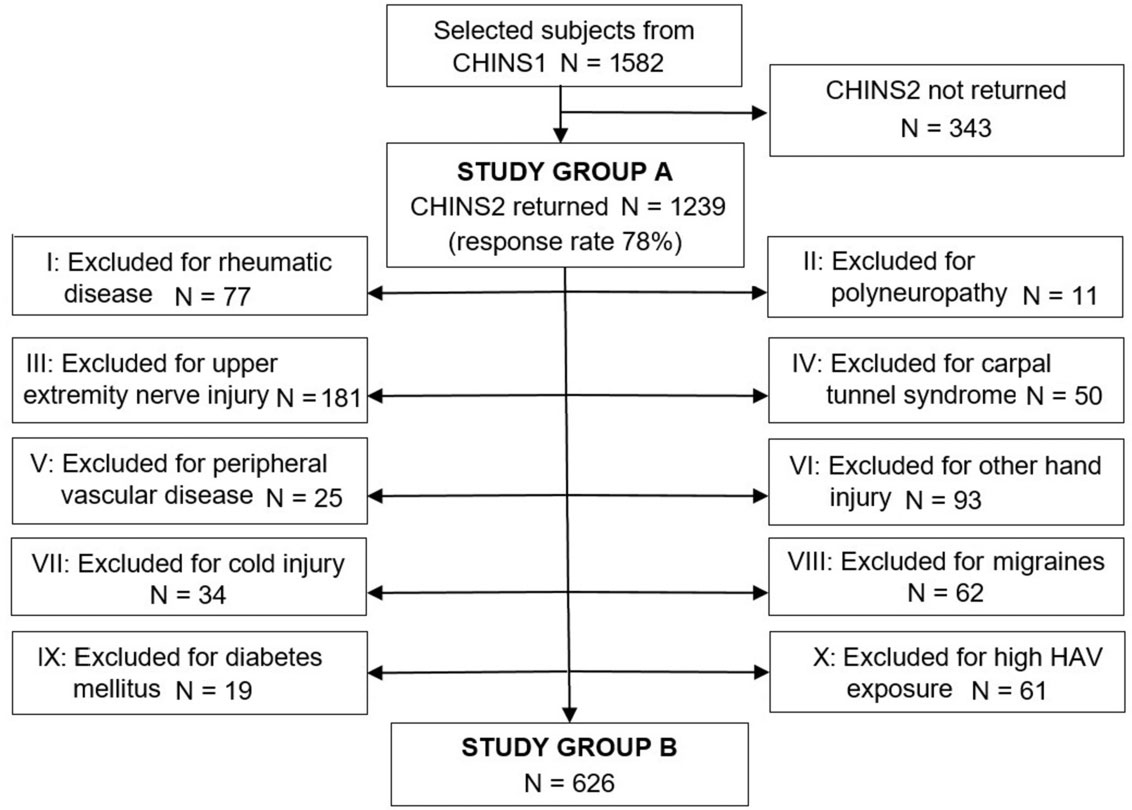
Data collection. Roman numerals describe the stepwise exclusion process from the healthy (Study Group A) to the even healthier subjects (Study Group B). CHINS 1-2: Cold and Health in Northern Sweden questionnaire 1 and 2. HAV: hand-arm vibration.

Among the responding healthy subjects (Study Group A), a further exclusion process was performed to identify an even healthier population for subgroup analysis (Study Group B) (Figure 1). These additional exclusion criteria, that have previously been shown to be associated with reporting cold sensitivity (Novak, 2018; Stjernbrandt et al., 2018), were added in the same stepwise order as follows: rheumatic disease (e.g. systemic sclerosis or rheumatoid arthritis); polyneuropathy; upper extremity nerve injury (e.g. avulsion, cervical radiculopathy); carpal tunnel syndrome; peripheral vascular disease; other hand injury (e.g. fractures or lacerations); cold injury affecting the hands; migraines; diabetes mellitus; and high occupational exposure to HAV (recurrent occupational use of impact tools, such as rock drills and chipping hammers, or heavily vibrating tools, such as reciprocating saws and oscillating sanders).

### Statistical methods

Data were described as median values and interquartile ranges (IQR) for continuous variables (unless otherwise stated), and as numbers and percentages for categorical variables. Analysis of skewness and kurtosis, as well as the Shapiro–Wilk test, indicated that age and CISS data were not normally distributed, motivating the use of non-parametric tests. The Mann–Whitney *U* test was used for quantitative, and the chi-square test for categorical variables. Spearman’s rank correlation coefficient was calculated for age and CISS score. The upper 95th percentile for the total sum of the CISS score, and the mean + 2 SD, were both used to establish a cut-off for abnormal cold sensitivity. Missing data were excluded from analysis, and percentages presented as valid.

## Results

### Recruitment

Of the 1582 healthy subjects who were eligible for participation, 1239 had returned the questionnaire, yielding a response rate of 78% (Figure 1). The final study population (Study Group A) consisted of 463 men and 776 women, with a median age of 59 (IQR 16) and 52 (IQR 20) years, respectively. Body mass index was 26.4 (IQR 4.4), and 25.2 (IQR 6.0) kg/m^2^, respectively. Additional descriptive data can be found in Table 1.

**Table 1.** Descriptive characteristics for study participants (Study Group A).

Subject data	Men (*N* = 463)		Women (*N* = 776)	
	Number	%	Number	%
Daily smoking	39	9	68	9
Daily use of snuff	110	24	43	6
Hypertension	128	29	162	22
Angina pectoris	14	3	15	2
Myocardial infarction	17	4	10	1
Stroke	5	1	8	1
Asthma	41	9	90	12
Chronic obstructive pulmonary disease	3	1	12	2
Diabetes mellitus	23	5	37	5
Joint disease	30	7	82	11
Migraines	24	5	87	12
Frostbite affecting the hands	33	7	34	4
Rheumatic disease	24	5	53	7
Polyneuropathy	5	1	8	1
Upper extremity nerve injury	91	20	120	16
Peripheral vascular disease	15	3	26	3
Carpal tunnel syndrome	31	7	68	9
Outdoor work ≥ ¼ of time	186	41	79	10
Work handling cold objects	162	35	62	8
Any occupational HAV exposure	207	47	52	7
Heavy occupational HAV exposure^a^	112	25	16	2

HAV: hand-arm vibration.

a Recurrent occupational use of impact tools or heavily vibrating tools.

### Results of scoring on the CISS questionnaire (Study Group A)

**Figure 2.**
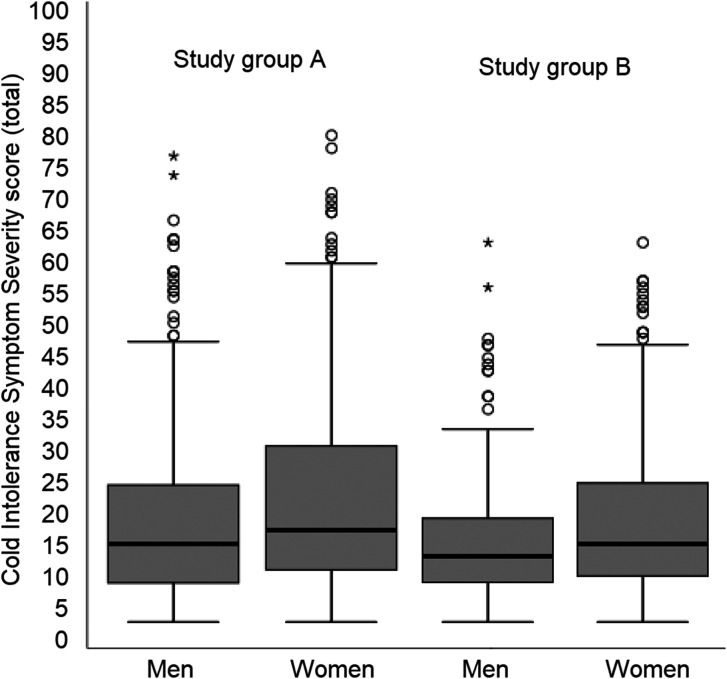
Box plots showing total Cold Intolerance Symptom Severity (CISS) scoring for the healthy (Study Group A) and the even healthier subjects (Study Group B), separated by gender. The grey boxes represent interquartile ranges (IQR), the lines across the boxes the medians and the whiskers 1.5 × IQR. Open circles display outliers (beyond 1.5 × IQR), and stars extreme outliers (beyond 3.0 × IQR).

**Figure 3.**
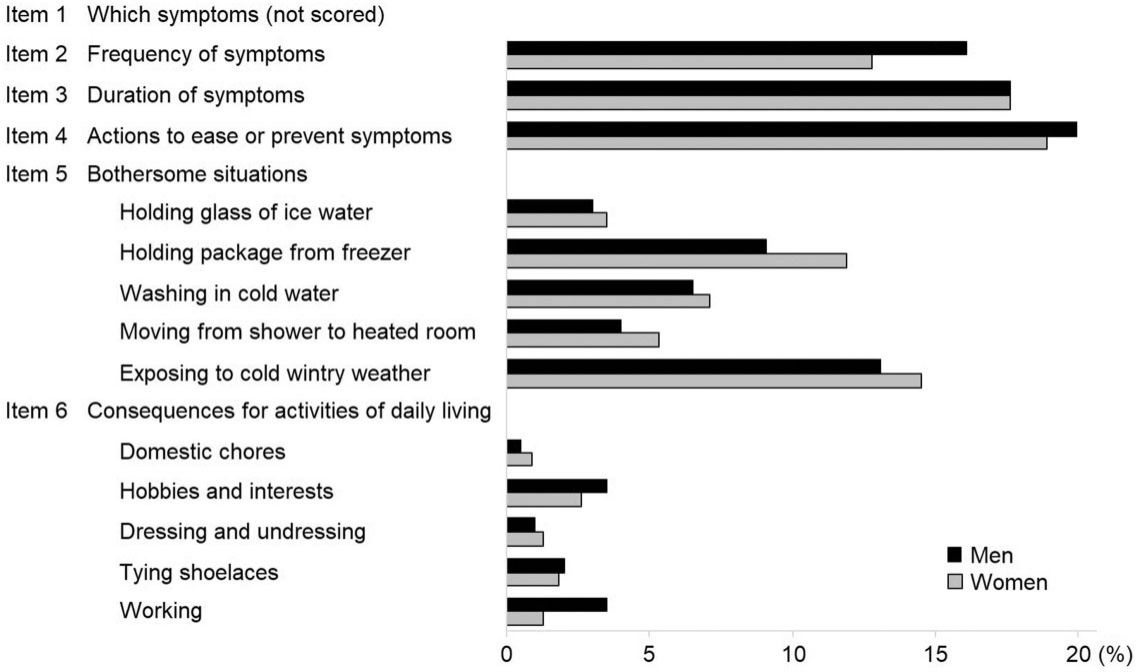
Distribution of Cold Intolerance Symptom Severity (CISS) scoring, presented as percentage of the total score in healthy subjects (Study Group A).

The median CISS score was 16 (IQR 15; range 4 to 76) for men and 18 (IQR 19; range 4 to 79) for women (Figure 2). The 95th percentile for the CISS score was 49.5 for men, and 53.0 for women, and the mean + 2 SD 47.2 and 51.1, respectively. The fourth CISS item (i.e. what actions were taken to ease or prevent cold-related symptoms) contributed most to the overall sum, constituting 20% of the sum in men, and 19% in women (Figure 3). Among bothersome situations (CISS item five), cold wintry weather outdoors was the largest contributor, rendering 13% of the CISS sum in men and 15% in women. The points scored for consequences for activities of daily living was lower, constituting 1% to 4% and 1% to 3% of the total sum, respectively. Missing data ranged from 5% to 14%, depending on CISS item.

### Effects of age and gender (Study Group A)

There was no significant difference in reporting CISS > 50 between men and women (*p* = 0.548). Age showed no significant effect on reporting CISS > 50 (*p* = 0.094 for men, and *p* = 0.375 for women). Spearman’s rank correlation coefficient for age and CISS score was 0.16 (*p* = 0.003) for men, and –0.04 (*p* = 0.295) for women.

### Sub-group analysis (Study Group B)

After excluding study participants with previous injuries and diseases, possibly affecting the risk of cold-related complaints, 626 subjects remained in Study Group B (Figure 1). In this group, the median CISS score was 14 (IQR 10; range 4–63) for men, and 16 (IQR 15; range 4–63) for women (Figure 2). The 95th percentile was 44.2 for men and 46.0 for women, and the mean + 2 SD 39.1 and 43.6, respectively. Spearman’s rank correlation coefficient for age and CISS score in Study Group B was 0.12 (*p* = 0.158) for men and –0.06 (*p* = 0.276) for women.

### Defining the threshold for cold sensitivity

Based on the results of the 95th percentile for men and women, we postulated that a score of 50 should be a suitable cut-off for abnormal cold sensitivity.

## Discussion

According to the CISS score, some degree of cold-related hand symptoms were common among healthy subjects in northern Sweden, with a 95th percentile for CISS scoring of 49.5 for men and 53.0 for women. Excluding subjects with a range of diseases and injuries known to be associated with cold sensitivity lowered the 95th percentile by a mere 5.3 and 7, respectively. Age did not appear to have a major influence on CISS scoring.

Our results support the findings by Carlsson et al. (2010a), who originally suggested CISS > 50 as a clinical cut-off for pathological cold introlerance. Although the score of 50 is much higher than the score of 30, as previously proposed by Ruijs et al. (2006), it is unlikely that different statistical approaches could explain the large difference in outcome, for example, basing the cut-off on the upper 95th percentile, or the mean + 2 SD. The population in the present study had a higher median age and a larger percentage of female participants than the previous two studies, but all three studies failed to demonstrate a major effect of age and gender on CISS scoring among healthy subjects. One possibility is that the differences in scoring might be due to effects of translating the CISS questionnaire from English to Dutch and Swedish. However, the Swedish version has been carefully tested for uniformity with the original version (Carlsson et al., 2008).

Another possibility is that selection effects in the recruitment of healthy subjects have affected the outcome. This was however disputed by the fact that the subgroup analysis in the present study did not reveal a very large effect of excluding for injuries and diseases (Figure 2). Rather, it is likely that Swedish subjects are more highly exposed to the cold climate, which may act both as a trigger for cold-related symptoms, but also as an aetiological factor in the development of neurovascular hand symptoms (Maricq et al., 1997; Stjernbrandt et al., 2018). This would explain why healthy Swedes of working age would report more cold-related complaints in their hands, although they are not injured. This view is also supported by the high scores on cold wintry weather as a bothersome situation (CISS item five) (Figure 3). However, it is interesting that the results of the previous Swedish reference material by Carlsson et al. (2010a) were so similar to those of the present study, given that the former was performed in the southernmost part of Sweden, and the latter in the much colder north. In comparison with the original four-grade categorization of the CISS questionnaire (Irwin et al., 1997), those who were deemed to have abnormal cold sensitivity in the present study would mostly have been classified as having severe, or extremely severe cold sensitivity, according to the categories used in the original article. This supports that the suggested cut-off in the present article is not likely to be set too low.

Defining the criteria of a pathological condition for a reference population remains challenging, for example to what length does one have to go to ascertain that they are indeed healthy? In the present study, the requirements for inclusion (Study Group A) were that the study participants did not have a previous history of cold sensitivity or Raynaud’s phenomenon, two major causes for high CISS scores. Reducing the population by approximately 50%, through a stepwise procedure of additional exclusion criteria (Figure 1), rendered an even more exclusive and likely healthier reference population (Study Group B). A difference in total CISS score between Study Group A and B was evident but rather small, indicating that the diseases and injuries included in this study did not have a very large impact on reporting cold-related complaints. The effects of age and gender in CISS scoring were also small and were not considered clinically relevant when deciding on a cut-off reference value.

There are several limitations in the present study. The population was dominated by women and had an age distribution that differed from that of the general population, since there was an overrepresentation of elderly. There was also an internal dropout due to missing data on different CISS items, which were likely not missing at random. This might limit the generalizability of the results. There are also a number of limitations within the CISS questionnaire itself. First, items two and three have no alternative for ‘not applicable’, meaning that the responder cannot get a score of less than two on either item, even if completely asymptomatic. Item four scores the alternative ‘other’ with a maximum of ten points, regardless of what is specified in free-form text. Item five constitutes half of the total score, which seems an unjust weighting in relation to the other items. Finally, question six asks for consequences for daily living, but uses activities not typically recognized to be a problem for subjects with cold sensitivity (e.g. ‘dressing and undressing’ or ‘tying shoelaces’), according to an interview study on subjects with cold sensitivity (Stjernbrandt et al., 2020). In addition, no information is gathered on the distribution of cold sensitivity, which means that CISS scoring is unchanged by describing symptoms in only one finger, compared with both whole arms, as long as the perceived consequences are the same. Finally, the questionnaire implies the assumption that an injury has occurred, preceding the onset of cold sensitivity. As has been shown previously, this is not always the case (Campbell and Kay, 1998; Stjernbrandt et al., 2020; Vaksvik et al., 2016).

However, one major strength is the size of the present study, compared with previously reported reference materials. There was also access to many important parameters other than the CISS scoring, such as previous diseases and injuries, and different external occupational exposures (e.g. cold and HAV). Data on anthropometry, tobacco habits and concurrent diseases corresponded with other Swedish studies (Eriksson et al., 2011; Lindberg et al., 2006), which serves as an indication that the present study included a representative sample of the population.

In conclusion, this study supports a CISS score above 50 as a cut-off for abnormal cold sensitivity, for use in clinical practice. For hand surgery patients with cold-related sequelae, this threshold could prove useful to guide further care. The same cut-off can be used for men and women, regardless of age.

## Supplemental Material

sj-pdf-1-jhs-10.1177_1753193421996221 - Supplemental material for Defining abnormal cold sensitivity using the Cold Intolerance Symptom Severity questionnaire: a population study Click here for additional data file. Supplemental material, sj-pdf-1-jhs-10.1177_1753193421996221 for Defining abnormal cold sensitivity using the Cold Intolerance Symptom Severity questionnaire: a population study by Albin Stjernbrandt, Ingrid Liljelind, Tohr Nilsson and Jens Wahlström in Journal of Hand Surgery (European Volume)
