# Supplementary material for: Defining abnormal cold sensitivity using the Cold Intolerance Symptom Severity questionnaire: a population study
Source: J Hand Surg Eur Vol. 2021 Mar 12;46(7):731–7. doi: 10.1177/1753193421996221 (PMC8377284; doi:10.1177/1753193421996221)
Supplement: sj-pdf-1-jhs-10.1177_1753193421996221 - Supplemental material for Defining abnormal cold sensitivity using the Cold Intolerance Symptom Severity questionnaire: a population study [file sj-pdf-1-jhs-10.1177_1753193421996221.pdf]

Table S1. The Cold intolerance Symptom Severity (CISS) questionnaire (Irwin et al., 1997).

**1. Which of the following symptoms of cold intolerance do you experience in your injured limb on exposure to cold? Pain, numbness, stiffness, weakness, aching, swelling, skin colour change (white/bluish white/blue)**	Not scored
**2. How often do you experience these symptoms?**	
Continuously/all the time	10
Several times a day	8
Once a day	6
Once a week	4
Once a month or less	2
**3. When you develop cold induced symptoms, on your return to a warm environment are the symptoms relieved?**	
Within a few minutes	2
Within 30 minutes	6
After more than 30 minutes	10
**4. What do you do to ease or prevent your symptoms occurring?**	
Take no special action	0
Keep hand in pocket	2
Wear gloves in cold weather	4
Wear gloves all the time	6
Avoid cold weather/stay indoors	8
Other (please specify)	10
**5. How much does cold bother your injured hand in the following situations?**	
Holding a glass of ice water	0–10
Holding a frozen package from the freezer	0–10
Washing in cold water	0–10
When you get out of a hot bath/shower with air at room temperature	0–10
During cold wintry weather	0–10
**6. Please state how each of the following activities have been affected as a consequence of cold induced symptoms in your injured hand and score each.**	

Domestic chores	0–4
Hobbies and interests	0–4
Dressing and undressing	0–4
Tying your shoe laces	0–4
Your job	0–4
**CISS total**	4-100
